# Dimerization‐dependent NOTCH receptor transactivation unveils a class of highly selective NOTCH signalling inhibitors

**DOI:** 10.1111/febs.70312

**Published:** 2025-11-01

**Authors:** Xinxin Liu, Haijiang Wang, Gunja Mishra, Lin‐Ting Wu, Chao Li, Maarten van Dinther, Jin Liu, Manuel A.F.V. Gonçalves, Peter ten Dijke, David A. Baker

**Affiliations:** ^1^ Oncode Institute and Department of Cell & Chemical Biology Leiden University Medical Center (LUMC) The Netherlands; ^2^ Department of Cell & Chemical Biology Leiden University Medical Center (LUMC) The Netherlands; ^3^ Department of General Surgery The First Affiliated Hospital, Xi'an Jiaotong University China

**Keywords:** acute lymphoblastic leukaemia, NOTCH receptor inhibitors, NOTCH signalling, peptide‐based inhibitors, targeted therapy

## Abstract

The NOTCH pathway is an ancient, ubiquitously expressed cell signalling system that is essential for embryonic and postembryonic cell fate control and tissue growth. It is activated via intercellular interactions between receptors and ligands expressed by neighbouring cells. This releases the latent transcription‐promoting potential of the receptor through proteolytic cleavage of the intracellular domain, which translocates to the nucleus and directly stimulates target gene expression as part of a DNA‐bound transcription activation complex. To date, it has been assumed that this process involves interactions between ligand homomers and receptor homomers. In this study, we present several lines of evidence in support of the view that NOTCH receptor dimerization/oligomerization (hereafter referred to as dimerization) could be necessary for the transactivation of receptor signalling. We show that (1) NOTCH receptors can efficiently self‐associate, which is mediated via a short motif found in the negative regulatory region (NRR) of the extracellular domain; (2) deletion of this motif ablates receptor homodimerization and blocks receptor transactivation; and (3) short peptides specifically targeting the dimerization motif similarly block receptor dimerization and receptor transactivation. Since the NOTCH pathway is corrupted in a wide range of disorders, the newly identified mechanism of NOTCH receptor transactivation presented here, and the discovery of a unique class of NOTCH signalling inhibitors, potentially reveals new therapeutic avenues to treating these diseases. In this light, a proof‐of‐concept study centred on T‐cell acute lymphoblastic leukaemia (T‐ALL) is presented.

## Graphical abstract

NOTCH signalling is indispensable for tissue homeostasis and, consequently, corruption of its normal function promotes numerous diseases, including cancer. However, the development of targeted therapies has been hampered by inefficacy and overt toxicity. Here, we show that NOTCH receptor dimerization is necessary for receptor transactivation, which has enabled the discovery of a previously unknown class of highly selective NOTCH signalling inhibitors that could underpin novel therapeutic approaches to treating NOTCH‐driven pathologies.

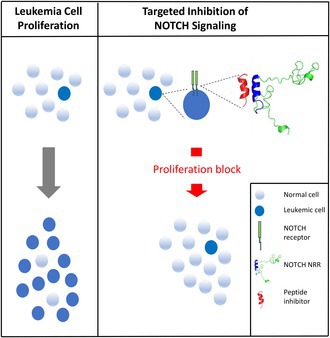

**Abbreviations**
BSA: bovine serum albumen
CADASIL: cerebral autosomal dominant arteriopathy with subcortical infarcts and leukoencephalopathy
DAPT: N‐(N‐(3,5‐difluorophenacetyl)alanyl)phenylglycine tert‐butyl ester
DLL: DELTA‐LIKE
DMEM: Dulbecco's Modified Eagle Medium
ECD: Extracellular domain
EGF: epidermal growth factor
EMT: epithelial to mesenchymal transition
FBS: fetal bovine serum
GFP: green fluorescent protein
GSI: gamma secretase inhibitor
JAG: JAGGED
LNR: LIN‐12/ NOTCH repeats
N: NOTCH
NRR: negative regulatory region
PBS: phosphate buffered saline
PLA: proximity ligation assay
RPMI: Roswell Park Memorial Institute
SEC‐MALS: size exclusion chromatography with multi‐angle static light scattering
SPR: surface plasmon resonance
T‐ALL: T‐cell acute lymphoblastic leukaemia

## Introduction

The ubiquitous NOTCH signalling system is one of the major cell signalling pathways that play a central role in animal tissue biology [1, 2]. It was first identified in *Drosophila* during the early part of the last century, and so named because variants caused a characteristic NOTCH in the fruit fly wings [3]. Since then, the molecular details of its basic mechanisms, and its pivotal role in tissue development and maintenance, have been comprehensively dissected in a wide range of species covering the span of metazoan evolution from insects to humans [4, 5]. The core pathway comprises type 1 transmembrane receptors and type 1 transmembrane ligands. Invertebrates, such as *Drosophila*, possess a single NOTCH receptor family member controlled by two ligands, whilst vertebrates encode up to four distinct receptor types (NOTCH1‐4) and five different ligands: JAGGED (JAG)1, JAG2, DELTA‐LIKE (DLL)1, DLL3 and DLL4 [6, 7]. The overall architecture of the receptors, from *Drosophila* to human, has remained relatively unchanged. Mature heteromeric NOTCH receptors are large proteins (upwards of 300 kDa) and consist of an extracellular ligand binding domain coupled to a membrane‐anchoring intracellular portion, which encodes the intrinsic NOTCH receptor transcription activation potential. The extracellular domain (ECD) constitutes the bulk of the mass (in the range of 250 kDa) composed of linked epidermal growth factor (EGF)‐like domains: NOTCH1 and NOTCH2 encode 36 such domains, NOTCH3 has 34 domains, and NOTCH4 has 29 domains [8]. Structural studies utilizing specific ECD tracts suggest that receptor EGF‐like repeats minimally function as sites of interaction with the EGF‐like repeats, and other distinct domains, of the NOTCH ligands, and a number of such sites have been mapped for a subset of receptor–ligand combinations [9, 10, 11, 12].

The EGF‐like domains are connected to a juxta‐membrane negative regulatory region (NRR) formed of the heterodimerisation domain (through which the extracellular and intracellular portions of the receptor are linked) and three contiguous LIN‐12/NOTCH repeats (LNRs). This region functions as an essential component of the mechanosensory activity reported to underlie ligand‐dependent receptor activation [13]. Operationally, in the absence of ligand, it is understood to adopt an autoinhibitory conformation and upon ligand binding pulling forces expose a buried ADAM metalloprotease S2 cleavage site, which initiates the cascade of proteolytic events that ultimately lead to activation of NOTCH receptor target genes [14, 15, 16, 17]. Interestingly, detailed structural analyses of the isolated NRR region of different NOTCH receptor paralogues revealed a propensity to form inverted‐mirror‐image dimers stabilized by contacts between common, conserved helices at the interface, which could reinforce the autoinhibitory receptor ‘off‐state’ [18, 19, 20]. These analyses also provided important insights into the mechanistic basis of aberrant NOTCH receptor activation in diseases such as T‐acute lymphoblastic leukaemia (T‐ALL) [21], which is characterized by activating mutations that cluster in the NRR of NOTCH1 [22, 23, 24, 25]. Indeed, the central importance of NOTCH in normal tissue development and homeostasis is revealed by the broad spectrum of diseases in which normal NOTCH signalling is corrupted [2, 8]. In addition to T‐ALL, NOTCH plays an important role in oncogenesis in a wide spectrum of other tumours including lymphoma, prostate cancer and colorectal cancer [26, 27, 28]. Disease‐promoting alterations of NOTCH receptor function are also found in nontumour‐related diseases; for example, cerebral autosomal‐dominant arteriopathy with subcortical infarcts and leukoencephalopathy (CADASIL) and pulmonary arterial hypertension have been linked to NOTCH3 mutations/dysfunction [29, 30], and bicuspid aortic valve disease has been linked to mutations of NOTCH1 [31].

Consequently, during the last two decades, there has been a global effort to identify molecules that can specifically block aberrant NOTCH signalling, which has yielded a number of modalities including antibodies that directly target the receptor and its ligands, small‐molecule inhibitors of regulatory enzymes, most notably gamma secretase inhibitors (GSIs), and inhibitors of post‐translational modifications, such as glycosylation and acetylation, as well as microRNAs [8, 32, 33, 34, 35, 36, 37]. GSIs were the first NOTCH receptor inhibitors to be tested clinically and have remained at the forefront of efforts to devise a treatment of clinical utility [32, 33, 34, 35, 36, 37]. However, a major drawback of such approaches is the overt toxicity that results from the large number of substrates (in excess of 90), in addition to the majority of NOTCH receptor paralogues, which are targeted by these enzymes. Moreover, during tissue growth and differentiation, distinct NOTCH paralogues perform specific functions in an exquisitely regulated, spatially and temporally restricted manner, which can be either stimulatory or inhibitory depending on the context. Likewise, depending on the tumour cell or its function in the tumour microenvironment, NOTCH signalling can be either tumour‐promoting or tumour‐suppressive [38], and for these reasons, pan‐NOTCH signalling inhibition could yield unpredictable and unfavourable outcomes.

A potential solution to these currently intractable problems would be the development of highly selective inhibitors of NOTCH receptor activation, which can selectively and separately target each NOTCH paralogue. Here, we have taken a biochemical approach to re‐examine NOTCH receptor transactivation. Whereas the transcriptionally active cleaved NOTCH intracellular domain has been shown to assemble on DNA as a dimeric complex [39, 40, 41], in the absence of alternative models, it has generally been assumed that the initiation of signalling at the cell surface is executed via essentially monomeric receptor/ligand interactions. We present evidence that receptor dimerization is necessary for transactivation. This finding has enabled the identification of novel, highly specific peptide inhibitors that block both receptor dimerization and the resultant receptor transactivation. These first‐in‐class inhibitors could unveil a new approach to treating diseases in which NOTCH signaling is disrupted.

## Results

### A helical motif in the NOTCH3 (N3) NRR is necessary and sufficient for receptor dimerization

**Fig. 1:**
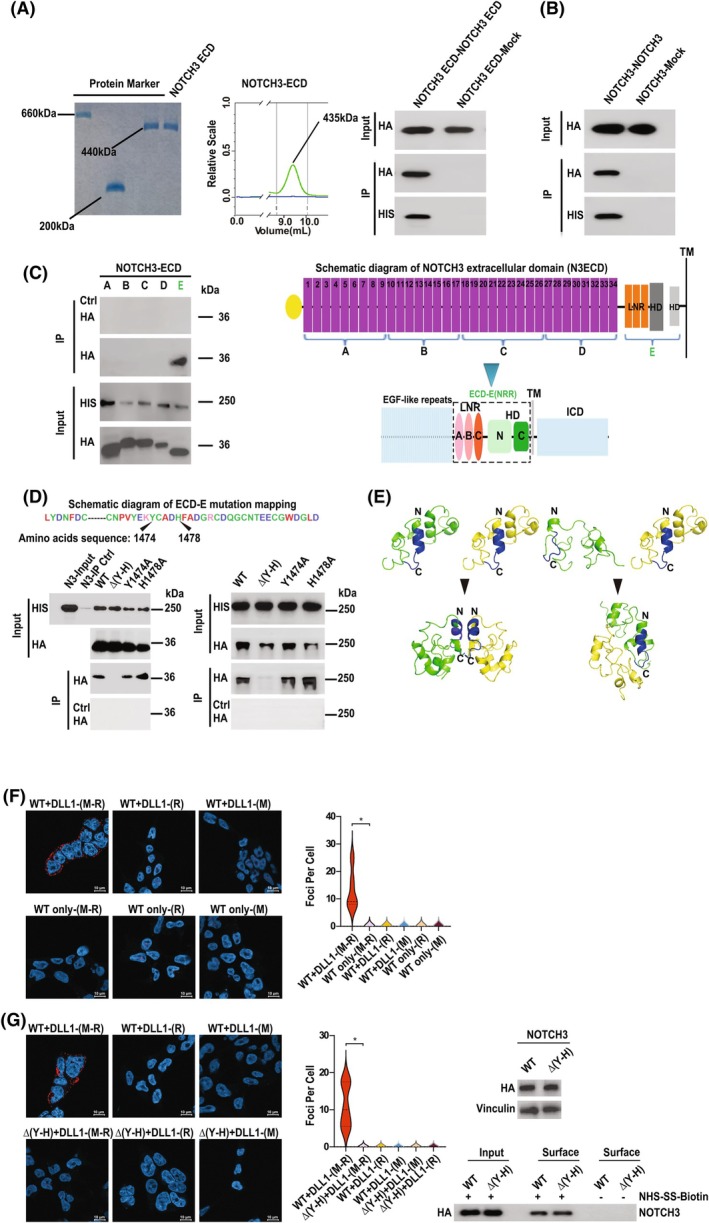
A short helical motif in the NRR of NOTCH3 (N3) is necessary and sufficient for receptor dimerization/oligomerization. (A, B) N3 receptor homodimerization *in vitro* (A) and in 293 T cells (B). (A) Left panel: Non‐denaturing acrylamide gel electrophoresis of purified recombinant HIS epitope‐tagged N3 extra cellular domain (ECD). Molecular weight standards used: thyroglobulin (660 kDa), apoferritin (440 kDa), beta‐amylase (200 kDa). Middle panel: SEC‐MALS analysis of purified N3 ECD. Right panel: Purified recombinant N3 ECD was incubated with HA epitope‐tagged N3‐ECD produced by *in vitro* translation. Complexes were resolved by immunoprecipitation and receptor‐receptor interactions were determined by Western blotting using the shown antibodies (*n* = 3). (B) The indicated combinations of HA and HIS epitope‐tagged versions of full‐length N3 were transfected into 293 T tissue‐culture cells. Complexes were resolved by immunoprecipitation and visualized with the shown antibodies (*n* = 3). (C) Biochemical mapping of the N3 dimerization motif. The indicated HA epitope‐tagged N3 domains were cotransfected into 293 T cells with HIS epitope‐tagged N3 ECD. A schematic representation of the constructs highlights the epidermal growth factor (EGF) repeats, the LIN‐12/NOTCH repeats (LNR), the heterodimerization domain (HD), the transmembrane domain (TM) and the intracellular domain (ICD). Complexes were resolved by immunoprecipitation and visualized by western blotting with the highlighted antibodies (*n* = 3). (D) Biochemical fine‐mapping of the N3 dimerization motif. Left panel: The indicated mutations were engineered in the E‐ECD construct (highlighted in green in Fig. 1C). The conserved sequence of the mutated motif is shown. HA epitope‐tagged versions of these constructs were cotransfected, with HIS epitope‐tagged full‐length N3, into 293 T cells. Complexes were resolved by immunoprecipitation and visualized by western blotting with the highlighted antibodies. Right panel: the same as the left panel except mutations were introduced into HA epitope‐tagged full‐length N3 (*n* = 3). (E) *In silico* modelling of a N3‐negative regulatory region (NRR) homodimer encompassing the LIN‐12/NOTCH repeats (LNR)2 and LNR3 domains of the NRR (amino acids 1428–1505) in the presence (LEFT) or the absence (RIGHT) of the dimerization motif. Modelling was performed using alphafold3, and predicted structures were analysed using pymol. Orientation (N‐ and C‐termini) is denoted by N and C. The helix encompassing the core dimerization motif is highlighted (blue). (F, G) Proximity Ligation Assays (PLA) for the detection of NOTCH receptor dimerization in cells. (F) Representative images of PLA (see the Methods section) performed on 293 T cells stably expressing HA epitope‐tagged NOTCH3 in the presence or absence of 2 μg·mL^−1^ recombinant human DLL1. Relative amounts of detectable receptor dimerization per cell is presented graphically (an average of 100 cells were scored in each experiment). Scale bar = 10 μm (*n* = 3). (G) Representative images of PLA (see the Methods section) performed on 293 T cells stably expressing either HA epitope‐tagged NOTCH3 or HA epitope tagged NOTCH3Δ(Y–D), which lacks the dimerization motif. Experiments were performed in the presence of 2 μg·mL^−1^ recombinant human DLL1. Relative amounts of detectable receptor dimerization per cell is presented graphically (an average of 100 cells were scored in each experiment). Scale bar = 10 μm. Total NOTCH3 receptor levels were determined by western blotting using the indicated antibody, and relative receptor cell surface expression was determined by biotin labelling (see the Methods section). Experiments described in the figure were performed three times. The statistical hypothesis tests were performed using Student's *t*‐test. We defined *P* < 0.005 as a significant difference (*), and *P* ≥ 0.005 as not significant (n.s.).

Several elegant structural studies of isolated protein domains have identified the NRR of the NOTCH receptor ECD as a potential interface via which NOTCH receptors might associate [18, 19, 20, 21]. To further dissect NOTCH receptor self‐association, we initially focussed on NOTCH3 (N3). Figure 1A,B show that the N3 ECD and full‐length N3 could efficiently self‐associate *in vitro* (Fig. 1A) and in tissue culture cells (Fig. 1B), respectively. For *in vitro* experiments (Fig. 1A), we purified the complete (recombinant) N3 ECD from human tissue culture cells (glycomic and proteomic mass spectrometry analyses revealed that the protein was glycosylated and > 95% pure). It is notable that both size exclusion chromatography with multi‐angle static light scattering (SEC‐MALS) analysis of the N3 ECD, as well as the electrophoretic mobility of the protein (under nondenaturing conditions), suggested that the N3 ECD formed a homodimer (which can be disrupted pharmacologically—see Fig. 3H). To identify those domains potentially responsible for this interaction, we performed a comprehensive mapping analysis. Whilst the EGF‐like repeats do not appear to mediate receptor‐receptor binding, in agreement with previous studies [18, 19], Fig. 1C shows that the membrane‐proximal region encompassing the NRR interacted with the full‐length N3 ECD. The NRR of N3 is highly conserved over evolutionary time and shares significant structural identity with other NOTCH receptor family members (Fig. 1C). Both earlier structural analyses of the isolated NRR domain [18, 19] and *in silico* modelling (Fig. 1E) have highlighted a core helical motif, which could mediate self‐association of the full‐length receptor. To further define the potential functional significance of this motif, we engineered specific mutations of this sequence. Figure 1D shows that single point mutations of the putative dimerization interface were insufficient to disrupt self‐association, whereas a small deletion of the helix completely abrogated self‐association either of the N3 ECD or the N3 full‐length protein. Interestingly, *in silico* modelling of the NRR region revealed that the helix could form a core dimerization interface when monomers associate in a head‐to‐head orientation (Fig. 1E), and the overall structural integrity of the complex is predicted to be lost following deletion of the helix (Fig. 1E). To corroborate the presented biochemical evidence, we performed proximity ligation assays (PLA) to test receptor dimerization in cells. Figure 1F,G demonstrate that stable NOTCH receptor dimerization was triggered in response to ligand (Fig. 1F) and that deletion of the dimerization motif, which had no detectable effect either on overall receptor expression levels or cell surface expression, abolished this response (Fig. 1G).

Together, these findings show that a short helical motif in the NRR of N3 is necessary for receptor dimerization.

### N3 receptor self‐association mediates ligand‐dependent receptor transactivation

**Fig. 2:**
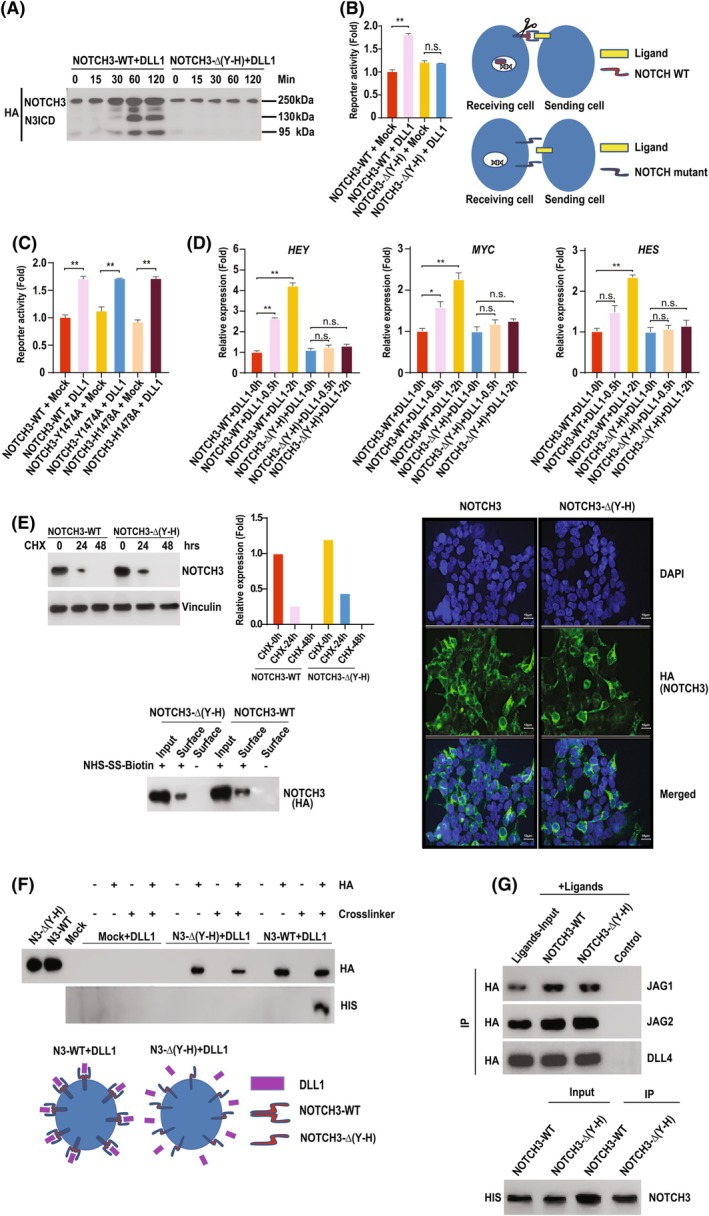
N3 dimerization mediates N3 receptor transactivation. (A) 293 T cells stably expressing HA epitope‐tagged wild‐type N3 or N3 lacking the dimerization motif, NOTCH3Δ(Y–H), were incubated in the presence or absence of recombinant DLL1 for the indicated time course. Receptor cleavage was monitored by western blotting using an HA antibody. Full length and cleaved forms of the N3 receptor are highlighted. (B) Luciferase reporter assays were performed as described in the Methods section, and illustrated schematically. The statistical hypothesis tests were performed using Student's *t*‐test. Values represent the mean ± SD. We defined *P* < 0.001 as a highly significant difference (**), *P* < 0.005 as a significant difference (*), and *P* ≥ 0.005 as not significant (n.s.) (*n* = 3). (C) Point mutations in the dimerization motif of the negative regulatory region (NRR), which do not block receptor dimerization, exhibit normal ligand‐dependent receptor transaction. Luciferase reporter assays were performed as described in the Methods section. The statistical hypothesis tests were performed using Student's *t*‐test. Values represent the mean ± SD. We defined *P* < 0.001 as a highly significant difference (**), *P* < 0.005 as a significant difference (*), and *P* ≥ 0.005 as not significant (n.s.) *n* = 3). (D) 293 T cells stably expressing the indicated N3 constructs were cultured in the presence or absence of recombinant DLL1 for the indicated time course. Endogenous gene expression levels were determined by Real‐time qPCR (see the Methods section). The statistical hypothesis tests were performed using Student's *t*‐test. Values represent the mean ± SD. We defined *P* < 0.001 as a highly significant difference (**), *P* < 0.005 as a significant difference (*), and *P* ≥ 0.005 as not significant (n.s.) (*n* = 3). (E) Deletion of the N3 dimerization motif does not detectably compromise receptor stability, cell surface expression or subcellular localization. UPPER LEFT: N3 receptor protein stability assay. 293 T cells stably expressing either HA epitope‐tagged wild‐type N3 or N3 lacking the dimerization motif (NOTCH3Δ(Y–H)) were incubated in the presence or absence of 50 μg·mL^−1^ cycloheximide (Sigma) for the shown time‐course. Protein levels were determined by Western blotting with an HA antibody. LOWER LEFT: Cell surface expression of N3 receptors. 293 T cells stably expressing either HA epitope‐tagged wild‐type N3 or N3 lacking the dimerization motif (NOTCH3Δ(Y–H)) were labelled with biotin (see the Methods section). N3 receptors were immunopurified from cell lysates and visualized by western blotting with an HA antibody. RIGHT: Immunofluorescence staining using the indicated antibodies was performed on tissue culture cells stably expressing either wild‐type N3 or N3 lacking the dimerization motif (NOTCH3Δ(Y–H)), as previously described [42]. Scale bar = 10 μm (*n* = 3). (F) Cell surface ligand–receptor binding assays (depicted schematically) were performed as described in the Methods section. Cells stably expressing the indicated HA epitope‐tagged N3 receptors were incubated with or without the indicated HIS epitope‐tagged ligand. Complexes were resolved by immunoprecipitation and ligand–receptor binding was determined by western blotting using the indicated antibodies (*n* = 3). (G) The dimerization motif is not necessary for NOTCH ligand binding. The indicated combinations of HA epitope‐tagged NOTCH ligands and HIS epitope‐tagged full‐length N3 receptors were expressed in 293 T cells. Receptor–ligand complexes were resolved by immunoprecipitation and visualized with the shown antibodies (*n* = 3).

Next, we investigated the function of receptor self‐association. Figure 2 shows, by four different means, that receptor homodimerization is necessary for receptor transactivation. One, Fig. 2A shows that ligand‐dependent cleavage of N3 was abolished by deletion of the dimerization motif. Two (and in line with the results presented in Fig. 2A), quantitative luciferase reporter assays showed that ligand‐dependent transactivation of the NOTCH reporter is likewise abolished following deletion of the dimerization motif (Fig. 2B). Correspondingly, point mutations of the motif, which do not block receptor self‐association (Fig. 1D), had no discernible inhibitory impact on receptor‐dependent activation of the reporter (Fig. 2C). Three, consistent with Fig. 2B, the dimerization motif was necessary for ligand‐dependent activation of endogenous, downstream NOTCH target genes (Fig. 2D). It should be noted that neither receptor cell surface expression, global subcellular localization nor overall receptor protein stability was detectably altered by deletion of the dimerization motif (Fig. 2E; Figs 1G and 2F). Four, and in line with our PLA data (Fig. 1F,G), deletion of the dimerization motif blocked stable ligand–receptor interactions at the cell surface (Fig. 2F). The NRR is not itself a site of interaction for NOTCH ligands [9, 10, 11, 12] (Fig. 2G) suggesting receptor dimer formation is a pre‐requisite for higher affinity/stable trans ligand binding.

Collectively, these data suggest that receptor dimerization is necessary for NOTCH receptor transactivation, and that a short helical motif located in the NRR is necessary for this mechanism.

### Identification of peptides that selectively block NOTCH receptor activation

**Fig. 3:**
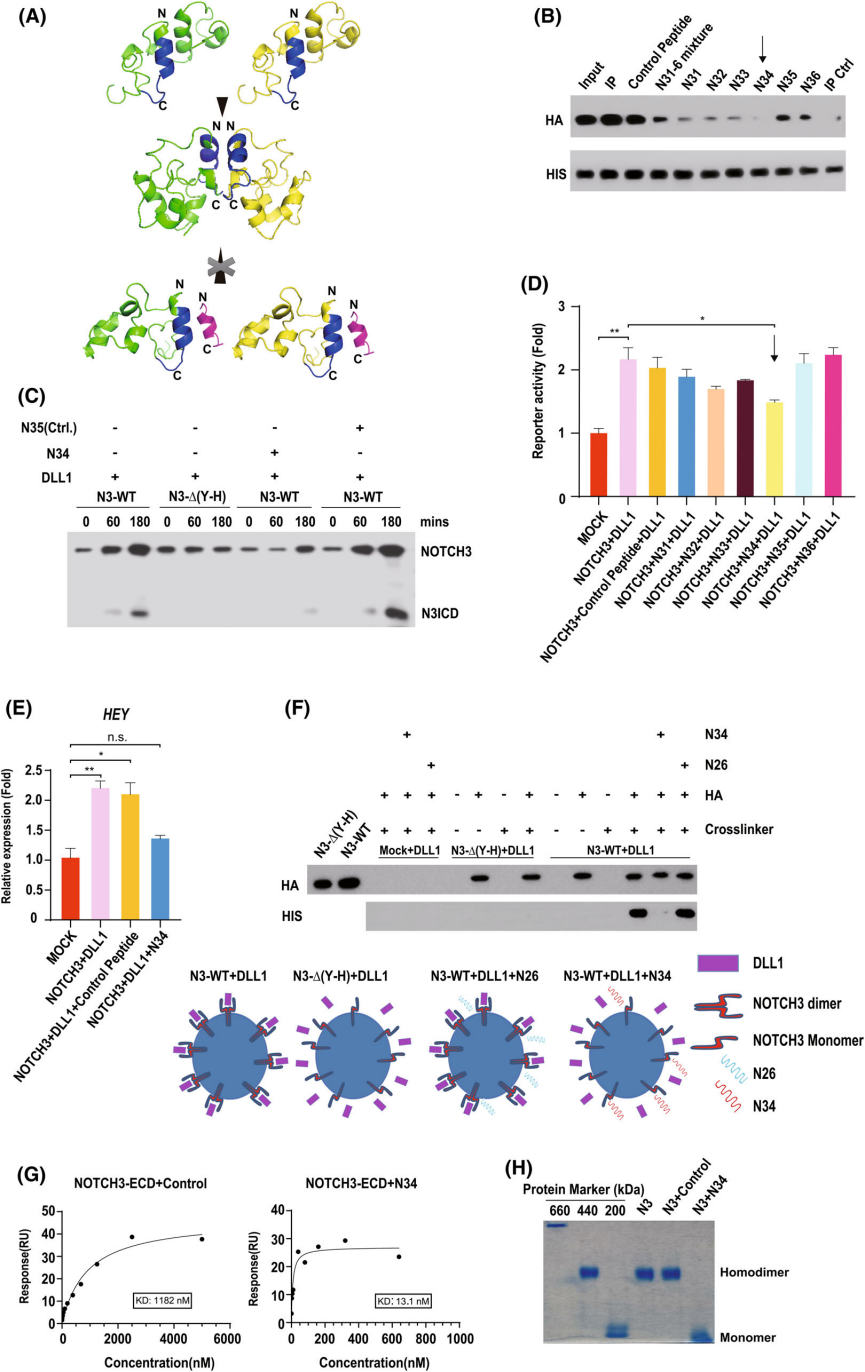
Identification of highly specific N3 inhibitors. (A) *In silico* modelling of N3‐negative regulatory region (NRR) dimer/peptide interactions. The N3 NRR homodimer encompasses the LIN‐12/ NOTCH repeats (LNR)2 and LNR3 domains of the NRR (amino acids 1428–1505). Modelling was performed using alphafold3, and predicted structures were analysed using pymol. Orientation (N‐ and C termini) is denoted by N and C. The N3 peptide (EKYCADHFADGR) (highlighted in magenta) was centred on the helix encompassing the core dimerization motif (blue) (see Table 1). (B) HIS epitope‐tagged N3 immunopurified from PEC.3.30 cells and immobilized on nickel beads was incubated with HA epitope‐tagged N3 in the presence or absence of the indicated peptides. The peptide selected for subsequent analyses is highlighted with an arrow. Receptor–receptor interactions were visualized by western blotting using the indicated antibodies (*n* = 3). (C) 293 T cells stably expressing HA epitope‐tagged wild‐type N3 or N3 lacking the dimerization motif, NOTCH3Δ(Y–H), were incubated in the presence or absence of recombinant DLL1, and the indicated peptides, for the shown time course. Receptor cleavage was monitored by western blotting using an HA antibody. Full length and cleaved forms of the N3 receptor are highlighted. (D) Luciferase reporter assays were performed, as described in the Methods section, in the presence or absence of the indicated peptides. The peptide selected for subsequent analyses is highlighted with an arrow. The statistical hypothesis tests were performed using Student's *t*‐test. Values represent the mean ± SD. We defined *P* < 0.001 as a highly significant difference (**), *P* < 0.005 as a significant difference (*), and *P* ≥ 0.005 as not significant (n.s.) (*n* = 3). (E) 293 T cells stably expressing HA epitope‐tagged N3 receptor were cultured (for 3 h) in the presence or absence of recombinant HIS epitope‐tagged DLL1 and the indicated peptides. Endogenous gene expression levels were determined by Real‐time qPCR (see the Methods section). The statistical hypothesis tests were performed using Student's *t*‐test. Values represent the mean ± SD. We defined *P* < 0.001 as a highly significant difference (**), *P* < 0.005 as a significant difference (*), and *P* ≥ 0.005 as not significant (n.s.) (*n* = 3). (F) Cell surface ligand–receptor binding assays were performed as described in the Methods section, and depicted schematically. Cells stably expressing the indicated HA epitope‐tagged N3 receptors were incubated with or without the indicated recombinant HIS epitope‐tagged ligand, in the presence or absence of the indicated peptides. Complexes were resolved by immunoprecipitation and ligand–receptor binding was determined by Western blotting using the indicated antibodies (*n* = 3). (G) A Surface Plasmon Resonance (SPR) analysis to determine the binding affinity of the N34 peptide (EKYCADHFADGR) and control peptide (EKYGGGHFADGR) to pure recombinant N3 ECD. Assays (as described in the Methods section) were performed three times and a representative example is shown. (H) Nondenaturing acrylamide gel electrophoresis of purified HIS epitope‐tagged N3 ECD incubated in the presence or absence of the N34 peptide or a control peptide (as described in G). Molecular weight standards used: thyroglobulin (660 kDa), apoferritin (440 kDa), beta‐amylase (200 kDa) (*n* = 3).

**Fig. 4:**
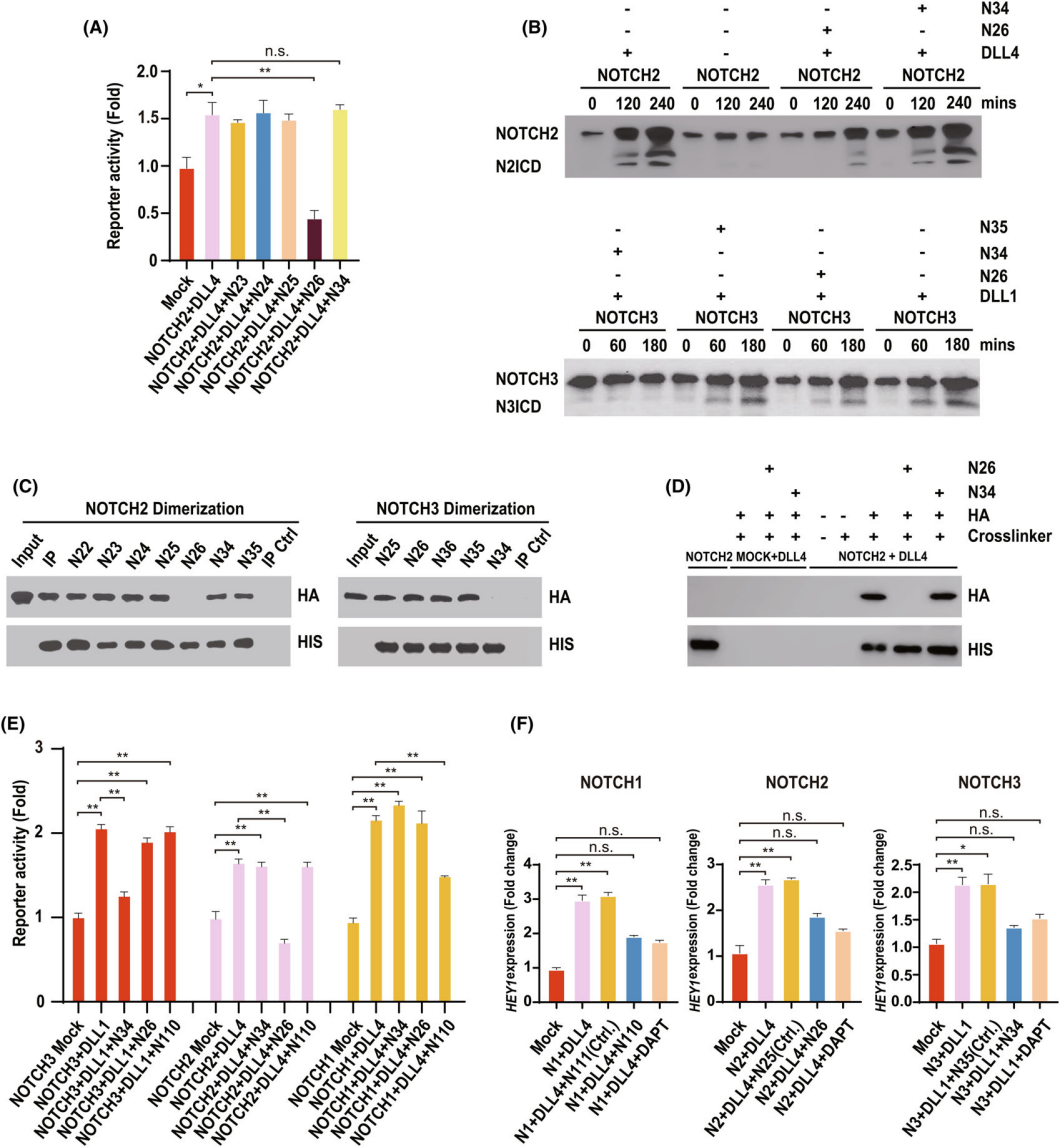
Identification of highly specific N2 and N1 inhibitors. (A) Luciferase reporter assays were performed as described in the Methods section, in the presence or absence of the indicated peptides. The statistical hypothesis tests were performed using Student's *t*‐test. Values represent the mean ± SD. We defined *P* < 0.001 as a highly significant difference (**), *P* < 0.005 as a significant difference (*), and *P* ≥ 0.005 as not significant (n.s.) (*n* = 3). (B) 293 T cells stably expressing HA epitope‐tagged N2 receptors (upper panel) or N3 receptors (lower panel) were incubated in the presence or absence of recombinant DLL1, and the indicated peptides, for the shown time course. Receptor cleavage was monitored by western blotting using an HA antibody. Full length and cleaved forms of the receptors are highlighted (*n* = 3). (C) Left panels: HIS epitope‐tagged N2 immunopurified from tissue culture cells and immobilized on nickel beads was incubated with HA epitope‐tagged N2 in the presence or absence of the indicated peptides. Receptor–receptor interactions were visualized by western blotting using the indicated antibodies. Right panels: The same experiments were performed, as described in the upper panels, using epitope‐tagged N3 in place of N2 (*n* = 3). (D) Cell surface ligand–receptor binding assays were performed as described in Fig. 3F (see the Methods section) (*n* = 3). (E) Luciferase reporter assays were performed as described in the Methods section. 293 T tissue culture cells stably expressing the indicated NOTCH receptors were stimulated with the indicated NOTCH ligands in the presence or absence of the indicated peptides. The statistical hypothesis tests were performed using Student's *t*‐test. Values represent the mean ± SD. We defined *P* < 0.001 as a highly significant difference (**), *P* < 0.005 as a significant difference (*), and *P* ≥ 0.005 as not significant (n.s.) (*n* = 3). (F) 293 T cells stably expressing the indicated NOTCH receptors were stimulated (for 3 h) by the indicated NOTCH ligands in the presence or absence of the indicated peptides or DAPT (20 μm). Endogenous *HEY* expression levels were determined by Real‐time qPCR (see the Methods section). The statistical hypothesis tests were performed using Student's *t*‐test. Values represent the mean ± SD. We defined *P* < 0.001 as a highly significant difference (**), *P* < 0.005 as a significant difference (*), and *P* ≥ 0.005 as not significant (n.s.) (*n* = 3).

We reasoned that if receptor dimerization is necessary for NOTCH transactivation, in common with deletion of the dimerization motif, pharmacological inhibition of the process would similarly block NOTCH signalling (shown schematically in Fig. 3A). To further validate our findings, we designed short (12–20 amino acid) peptides centred on the dimerization interface (Table 1). Figure 3 shows that a peptide, which selectively binds to the dimerization motif, could robustly inhibit receptor dimerization (Fig. 3B), ligand‐dependent receptor cleavage (Fig. 3C) and transactivation of both a NOTCH reporter (Fig. 3D) and downstream NOTCH target genes (Fig. 3E). Indeed, the peptide inhibitor suppressed NOTCH‐driven gene expression as efficiently as the generic gamma secretase inhibitor (GSI), DAPT (Fig. 4F). Finally, in common with deletion of the dimerization motif, the peptide efficiently blocked stable ligand–receptor binding at the cell surface (Fig. 3F). To estimate the binding affinity of the peptide for N3, we deployed surface plasmon resonance using pure, recombinant N3 ECD purified from human cells (Fig. 1). By these means, we determined the *K*_D_ to be in the low nm range (Fig. 3G). Significantly, a control peptide in which the amino acids composing the core of the dimerization motif (Figs 1 and 2) were mutated (to glycine) failed to bind efficiently to the N3 ECD, consistent with the idea that the motif is necessary and sufficient for peptide–receptor binding. In support of these findings, the pure recombinant N3 ECD homodimer was disrupted by the specific N3 peptide inhibitor (in the presence of the peptide inhibitor, the electrophoretic mobility corresponded to an N3 ECD monomer), but not a control peptide (Fig. 3H). In agreement with the view that the NRR mediates receptor transactivation, previous studies, although not explicitly linked to NOTCH receptor dimerization, showed that NOTCH activity could be blocked by antibodies directed against the NRR domain [43, 44].

**Table 1:** Sequence of peptides for targeting NOTCH receptor signalling.

Name	Amino acid sequence
**NOTCH 1**	
N12	PLYDQYCKDHFSDGH
N13	DQYCKDHFSDGHCDQ
N14	DQYCKDHFSDGH
N15	DQYCKDHFSD
N16	PLYDQYCKDHFSD
N17	PLYDQYCKDHFSDGHCDQ
N18	PLYDQYCKDHFSDGHCD
N19	PLYDQYCKDHFSDGHC
N110	PLYDQYCKDHFSDG
N111	PLYDQYCKDHFS
N112	PLYDQYCKDHF
N113	LYDQYCKDHFSDGH
N114	LYDQYCKDHFSDG
N115	LYDQYCKDHFSD
N116	YDQYCKDHFSDGH
N117	YDQYCKDHFSDG
N118	YDQYCKDHFSD
N119	DQYCKDHFSDG
N120	CNPLYDQYCKDHFSDG
N121	CNPLYDQYCKDHFSD
N122	CNPLYDQYCKDHFS
N123	NPLYDQYCKDHFSDG
N124	NPLYDQYCKDHFSD
N125	NPLYDQYCKDHFS
N126	NPLYDQYCKDHF
**NOTCH2**	
N21	KYDKYCADHFKDNHCDQ
N22	KYDKYCADHFKDNH
N23	DKYCADHFKDNHCDQ
N24	DKYCADHFKDNH
N25	DKYCADHFKDN
N26	KYDKYCADHFK
**NOTCH3**	
N31	PVYEKYCADHFADGRCDQ
N32	PVYEKYCADHFADGR
N33	EKYCADHFADGRCDQ
N34	EKYCADHFADGR
N35	EKYCADHFAD
N36	PVYEKYCADH

### NOTCH receptor signalling inhibitors are highly NOTCH paralogue‐specific

All NOTCH receptor family members harbour an NRR domain, and the overall structure of this domain (encompassing the dimerization motif) is conserved [18, 19, 20, 21] (Fig. 1C). However, strikingly, whilst the N3 peptide inhibited N3 receptor activity, it had little detectable effect on NOTCH2 (N2) receptor cleavage (Fig. 4B), N2 receptor dimerization (Fig. 4C), stable N2 receptor–ligand binding at the cell surface (Fig. 4D) or N2 receptor downstream gene activation (Fig. 4E). Since the N3 peptide displayed exquisite selectivity for N3 (and not N2 or N1—Fig. 4E), we designed peptides, centred on the N2 and N1 dimerization motifs, to specifically block N2 and N1 signalling. Figure 4 shows, like the observed N3 peptide‐mediated disruption of N3 signalling, that an N2 peptide, but not the N3 or the N1 peptide, strongly inhibited N2 receptor transactivation (Fig. 4A,E), ligand‐mediated N2 receptor cleavage (Fig. 4B), N2 receptor dimerization (Fig. 4C) as well as stable ligand‐receptor binding at the cell surface (Fig. 4D). In common with the specificity of the N3 inhibitor, these effects were N2 receptor‐specific, since the N2 peptide could not block N3 (or N1) receptor transactivation (Fig. 4E), N3 ligand‐dependent cleavage (Fig. 4B), N3 receptor dimerization (Fig. 4C) or N3 stable ligand–receptor binding at the cell surface (Fig. 3F). Likewise, a peptide based upon the N1 dimerization motif efficiently blocked N1 transactivation, but failed to detectably inhibit either N2 or N3 receptor activity (Fig. 4E). Moreover, we found that the NOTCH paralogue‐specific inhibitors could block expression of downstream NOTCH target genes as effectively as the generic GSI Inhibitor, DAPT (Fig. 4F).

In summary, the evidence presented here supports the view that receptor dimerization/oligomerization underpins ligand‐dependent receptor signalling. On this basis, we have identified a novel class of paralogue‐specific NOTCH receptor peptide inhibitors that block receptor self‐association and the resulting receptor transactivation.

### NOTCH1 signalling inhibitors selectively block constitutively active NOTCH1 signalling in T‐ALL cells and abrogate cell proliferation and tissue invasion

Since the NOTCH signalling network is frequently corrupted in a wide variety of diseases, a provocative implication of our findings is the potential utility of these peptides in selectively targeting aberrant NOTCH signalling, for example, in tumour cells. T‐acute lymphoblastic leukaemia (T‐ALL), a particularly aggressive variant of ALL, could represent a suitable starting point for such investigations [35]. Extensive genome‐wide analyses have pinpointed mutations of the NOTCH1 (N1) receptor as one of the most prevalent mutations found in the majority of patients [22, 23, 24, 25]. Many of these mutations have been reported to trigger illicit hyperactivation of the receptor, which could contribute to the evolution of the disease. Thus, a molecule that selectively targets activated N1 receptors would be expected to inhibit T‐ALL cell viability.

**Fig. 5:**
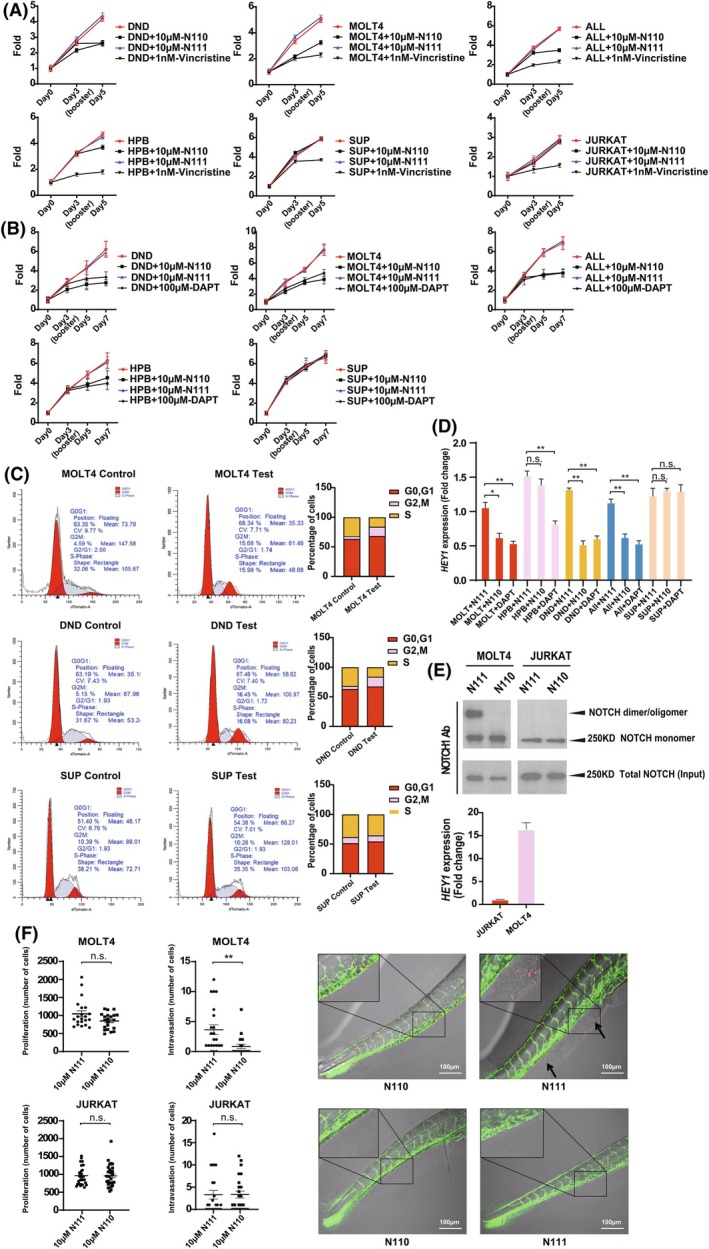
The N1 receptor inhibitor specifically blocks mutant hyperactive N1 receptor activation and cell proliferation of T‐ALL cell lines. (A, B) Cell viability assays of the indicated T‐cell acute lymphoblastic leukaemia (T‐ALL) cell lines (see the Methods section), were performed in the presence or absence of: (A) Vincristine, and (B) DAPT. Values represent the mean ± SD. of six replicates per data point. Fold change in cell viability is presented (*n* = 3). (C) Cell cycle status of the indicated T‐ALL lines was determined as described in the Methods section. (D) The indicated T‐ALL cell lines were cultured for 12 h in the presence or absence of the indicated treatments. Endogenous *HEY* gene expression levels were determined by Real‐time qPCR (see the Methods section). Values represent ± SD (*n* = 3). We defined *P* < 0.001 as a highly significant difference (**), *P* < 0.005 as a significant difference (*), and *P* ≥ 0.005 as not significant (n.s.). (E) UPPER PANEL: Endogenous N1 receptors expressed at the cell surface of MOLT4 cells and JURKAT cells were labelled with biotin (see the Methods section). N1 receptor complexes were immunopurified from cell lysates and visualized by Western blotting with an N1 antibody. LOWER PANEL: Endogenous *HEY* gene expression levels were determined by Real‐time qPCR (see the Methods section). Values represent ± SD. (*n* = 3). (F) Zebrafish embryo tumour cell invasion assay. Zebrafish embryos were injected with the indicated fluorescently labelled T‐ALL cell lines (red) in the presence or absence of the N1 peptide inhibitor or a control peptide. Blood vessels are labelled with green fluorescent protein (GFP). Cell invasion into nonvascular tissue (highlighted by arrows) and overall cell proliferation were quantified after 4 days (see the Methods section). Scale bar = 100 μm. The statistical hypothesis tests in this study were performed using Student's *t*‐test. We defined *P* < 0.001 as a highly significant difference (**), *P* < 0.005 as a significant difference (*), and *P* ≥ 0.005 as not significant (n.s.). Experiments were performed three times and data from a representative experiment are shown.

Figure 5 summarizes an analysis of six different patient‐derived T‐ALL cell lines. Four lines harbour N1 activating mutations: MOLT4 (also the related MOLT3 line was tested and yielded comparable results), ALL SIL, DND‐41 and HPB‐ALL. Two lines acted as controls: SUP‐T1 cells harbour an N1 translocation resulting in the loss of almost the entire ECD [45], which would preclude targeting of the dimerization motif in the NRR, and JURKAT cells encode N1 receptors, which are not hyperactive by comparison with the other T‐ALL lines. Figure 5A, B shows that the N1 peptide significantly inhibited the viability of the lines expressing N1 activating mutations but not the control cell lines. In each case, the effects were compared to the widely used chemotherapeutic reagent, vincristine (Fig. 5A), and high concentrations of the generic GSI, DAPT (Fig. 5B). Correspondingly, the peptide inhibitor disrupted cell cycle progression (Fig. 5C) of cell lines harbouring N1 mutations, but not the control cell line. Moreover, the peptide inhibitor blocked the expression of downstream N1 target genes to a degree comparable to the effects of DAPT (Fig. 5D). To gain additional insight into the mechanistic basis of activated N1 receptor targeting in T‐ALL cells, we labelled endogenous N1 expressed at the cell surface of MOLT4 cells. By these means, two populations of N1 were observed: an N1 species whose mass corresponds to monomeric N1, and a higher molecular weight species potentially corresponding to an N1 dimer/oligomer in line with the notion that activating mutations may trigger constitutive dimerization. Indeed, the expression of the downstream N1 target gene, *HEY*, is 10‐ to 15‐fold higher in MOLT4 cells compared to JURKAT cells (Fig. 5E). Compellingly, we found that whilst incubation of the cells with the control peptide had no discernible effect on either the pattern or the amount of detected N1 receptor, the N1 peptide significantly inhibited levels of the higher molecular weight N1 complex accordant with the notion that the peptide could function, at least in part, by blocking N1 self‐association and subsequent receptor activation (Fig. 5E). Under the same conditions, the peptide had no discernible effects on the N1 receptor in control JURKAT cells.

Although, historically, leukaemia has not been considered to be a metastatic disease, it exhibits an exceptional capacity to spread and proliferate, and also to invade a wide range of different tissues [46]. Zebrafish embryos are a tractable, *in vivo* system for monitoring this phenomenon and are especially suited to testing the effects of potential inhibitors of this process. Figure 5F shows that the N1 peptide, but not a similar control peptide, significantly and reproducibly blocked the invasion of human T‐ALL cells, harbouring N1 activating mutations, into nonvascular tissue, compared to T‐ALL cells that are unable to bind the peptide. Under these conditions, the peptides affected only cell intra‐/extravasation and not overall cell proliferation, as expected, due to the relatively low temperatures at which the embryos are incubated (Fig. 5F). This suggests that the peptide can suppress both activated NOTCH‐driven proliferation (Fig. 5A,B), and activated NOTCH‐driven tissue invasion, of tumour cells (Fig. 5F).

Collectively, these findings show that a highly specific N1 peptide can selectively inhibit mutant, constitutively active N1 receptors expressed by T‐ALL cells and thereby significantly abrogate their proliferative and invasive potential. It will be of considerable interest to determine if the peptide inhibitor can similarly block N1 activity in other tumour types, and components of the tumour microenvironment, such as tumour vasculature, and if our N2 and N3 peptide inhibitors can, likewise, specifically target and attenuate the illicit NOTCH signalling characteristic of a number of known diseases.

## Discussion

In this study, we provide the first evidence, to the best of our knowledge, that NOTCH receptor dimerization could mediate ligand‐dependent receptor transactivation. Our work has focussed on three human NOTCH paralogues (N1, N2 and N3); however, given the high degree of overall architectural/structural similarity between vertebrate and invertebrate NOTCH receptors and ligands, the mechanism could be universal. Indeed, electron microscopy studies of the ECD of *Drosophila* NOTCH indicated that it could form a dimer [47]. Importantly, structural studies of isolated NOTCH receptor domains identified specific sequences within the NRR of human NOTCH paralogues which could stabilize inverted‐mirror‐image NRR homodimers, which are thought to contribute to maintaining NOTCH in an ‘off‐state’ by blocking access of proteolytic enzymes to buried enzyme cleavage sites [18, 19]. Under normal physiological conditions, it is not clear whether, owing to topological constraints, such an upside‐down configuration is adopted by receptors expressed at the surface of the same cell or, in the context of the complete ECD, if a different noninverted orientation could be established. In this regard, *in silico* modelling of the NRR region (including only the LNR2 and LNR3 regions) predicted that monomers could associate in a head‐to‐head orientation and revealed that the core dimerization interface [18, 19, 20, 21] (Fig. 3A) could be corrupted upon loss of the dimerization motif (Fig. 1E). Our work suggests that NOTCH dimerization is a prerequisite both for efficient trans ligand binding and the resulting NOTCH receptor activation. There are several potentially important implications of this view of NOTCH signalling. To date, one major gap in NOTCH signalling knowledge, owing to the considerable technical difficulties of characterizing full‐length proteins *in vitro* and *in vivo*, relates to the precise nature of ligand, receptor and receptor–ligand complexes and how they interact to generate the broad spectrum of cellular processes governed by the NOTCH network. Recent evidence suggests that ligand homodimerization [48] and heterodimerization [49] could play a role in setting the balance between receptor transactivation and cis inhibition, which determines receptor signalling output. Receptor dimerization and how this mechanism is regulated could add a new point of control of NOTCH signalling output. Related to this, it is unknown whether trans receptor–ligand interactions differ conformationally from cis receptor–ligand interactions, and in this context, it could be of value to delineate if, and how, receptor dimerization might contribute to their distinct effects on receptor activity. Intriguingly, it has been shown that the cleaved NOTCH intracellular domain can associate with cofactors at consensus DNA‐binding sites as a dimeric complex that is indispensable for the normal functioning of certain tissues [39, 40, 41]. Plausibly, such a mechanism would be facilitated/fine‐tuned by dimerization‐dependent receptor activation at the cell surface.

Another significant implication of our findings is the unveiling of possible, previously overlooked biomedical/therapeutic avenues for understanding and treating a number of diseases. Given the centrality of NOTCH signalling in tissue development and homeostasis, and the discovery that it is frequently corrupted in a wide range of disorders [3, 8, 50], during the 40 years since the cloning and characterization of the NOTCH genes, there has been a global effort, particularly in the era of precision medicine and targeted therapies, to identify NOTCH signalling inhibitors [32, 34]. As alluded to earlier, GSIs represent a mainstay of current clinical trials though overt toxicity remains a serious issue. Our work offers a unique, alternative solution based upon our discovery that highly specific peptides, centred on the NOTCH dimerization interface, blocked NOTCH receptor signalling in a NOTCH paralogue‐specific fashion. In this paper, as a proof‐of‐principle test of this new approach, we examined the effects of the peptide on T‐ALL, an aggressive cancer distinguished by the presence of activating N1 mutations in the majority of patients [22, 23, 24, 25]. We showed that the N1 peptide blocked both tumour cell proliferation, tissue invasion and downstream gene activation as effectively as the potent generic GSI, DAPT. Strikingly, the peptide failed to affect T‐ALL cells, which exhibit nonhyperactivated levels of N1 receptor activity, suggesting it might specifically target aberrantly activated N1. These findings raise the possibility that a highly specific N1 peptide inhibitor could be utilized, either alone or in combination with other agents, to target T‐ALL tumours harbouring activating N1 mutations. Given the exquisite selectivity of the peptide, this approach could significantly limit the overt toxicity associated with currently used chemotherapies and generic GSIs. In addition to T‐ALL, corrupted NOTCH signalling has been reported to play a role in numerous other cancers [50], both in the biology of the tumour cells and the tumour stroma, such as the tumour vasculature [51, 52, 53]. Tumour angiogenesis is a critical step in tumourigenesis that enables cancer cells to escape metabolic constraints on cell proliferation by physically connecting the tumour to the circulation and thereby fuelling its growth and enabling metastasis [32, 54, 55, 56]. It differs quantitatively and qualitatively from normal angiogenesis; however, a complex interplay between different NOTCH receptors and ligands is presumed to play a fundamental role in tumour blood vessel sprouting. Since context‐dependent NOTCH receptor activation/inhibition can trigger opposing effects, a possible consequence of this is that generic NOTCH signalling inhibitors (such as GSIs) could provoke unexpected (and unwanted) outcomes. Directly targeting specific NOTCH paralogues, which play distinct roles in angiogenesis, for example, could potentially overcome these problems. Two additional facets of oncogenesis merit comment. First, on account of its established functions in the epithelial to mesenchymal transition (EMT) [57], activated NOTCH signalling has been implicated in tumour metastasis, including of prostate, breast and colorectal cancer [57, 58]. Furthermore, because NOTCH plays a pivotal role in programs of progenitor cell self‐renewal and differentiation, which drives tissue development and replenishment, evidence is emerging that active NOTCH signalling could underlie the enhanced resistance of cancer stem cells to drug treatments [59]. It will be of significant interest to investigate whether the NOTCH peptides can block these and other processes that are essential for the evolution of these malignancies, and also the broad range of non‐oncological diseases with causal links to dysregulated NOTCH receptor function, such as CADASIL [29], Hajdu‐Cheney syndrome [60] and pulmonary arterial hypertension [30].

## Materials and methods

### Cell culture, biochemistry and molecular biology

Human embryonic kidney (HEK) 293 T cells (RRID:CVCL_0063) were cultured in Dulbecco's Modified Eagle Medium (DMEM) (Gibco, Grand Island, NY, USA) supplemented with 10% fetal bovine serum (FBS) (Gibco). This cell line was obtained from the American Type Culture Collection (ATCC, Manassas, VA, USA). T‐ALL cell lines (kindly provided by Professor Jan Cools – VIB, Leuven): MOLT4 (RRID:CVCL_0013), ALL SIL (RRID:CVCL_1805), DND‐41 (RRID:CVCL_2022), HPB‐ALL (RRID:CVCL_1820), SUP‐T1 (RRID:CVCL_1714) and JURKAT (RRID:CVCL_0065), were cultured in Roswell Park Memorial Institute (RPMI) 1640 medium (Gibco) supplemented with 10% FBS (Gibco). Cells were maintained in a 5% CO_2_, 37 °C humidified incubator, tested monthly for mycoplasma contamination using a MycoAlert kit (Lonza, Walkersville, MD, USA), and checked for authenticity, in the last 3 years, by an in‐house service using short tandem repeat (STR) profiling. Transfections, lentivirus production and cell infections, western blotting and co‐immunoprecipitations have been described previously [59, 60]. All cell lysis buffers contained a cocktail of protease inhibitors (phenylmethylsulfonyl fluoride, trypsin inhibitor, pepstatin A, leupeptin, aprotinin). RNA isolation, first‐strand cDNA synthesis and analysis of expression of transcripts by quantitative (q) PCR were performed as described previously [61, 62]. The following primer sets were used (5′ to 3′ orientation): *HEY*1 FOR: AGGCTGGTACCCAGTGCTT; *HEY*1 REV: GCATTCCCGAAATCCCAAACT; *HES1* FOR: AAGAAAGATAGCTCGCGGCA; *HES1* REV: CGGAGGTGCTTCACTGTCAT; *MYC* FOR: TGAGGAGACACCGCCCAC; *MYC* REV: CAACATCGATTTCTTCCTCATCTTC. All qPCR values were averaged relative to the control genes, TATA‐binding protein (*TBP*), signal recognition particle receptor (*SRPR*) and calcium‐activated neutral proteinase 1 (*CAPNS1*). For each data point, PCRs were performed in triplicate, and error bars show standard deviations from the mean. Experiments were performed at least three times. The statistical hypothesis tests in this study were performed using Student's *t*‐test. We defined *P* < 0.001 as a highly significant difference (**), *P* < 0.005 as a significant difference (*), and *P* ≥ 0.005 as not significant (n.s.). Unless otherwise stated, Notch peptides were used at a concentration of 10 μm, which was sufficient to elicit a maximal inhibitory effect in most assays. Unless otherwise stated, all cDNAs were fused in‐frame with a C‐terminal FLAG, HIS or HA epitope tag and were cloned into the pLV lentiviral vector and pCS2 expression plasmid. Expression of these proteins was determined using antibodies directed against either the coding region of the protein or the epitope tag. Mutants were generated by site‐directed mutagenesis using Phusion High‐Fidelity DNA polymerase (Thermo Fisher, Waltham, MA, USA). All constructs were verified by Sanger sequencing (Macrogen, Amsterdam, Netherlands).

### Recombinant protein production/*in vitro* protein : protein interactions

Domains for recombinant protein production were cloned into the pET 28a vector in‐frame to an N‐terminal HIS_6_ epitope. His epitope‐tagged proteins were manufactured in *Escherichia coli*, BL21(DE3). Following sonication (Misonix Sonicator 3000) in 3 mL ice‐cold buffer/50 mL bacterial culture (150 mm NaCl, 2.7 mm KCl, Na_2_HPO_4_, KH_2_PO_4_, 20 mm imidazole, 10 mm β‐mercaptoethanol), proteins were purified onto 50 μL of Nickel agarose beads (Qiagen, Hilden, Germany) by 3 h rolling at 4 °C. Beads were washed in 10 × 1 mL of the same buffer. Protein yields were determined by Bradford assay (Bio‐Rad, Hercules, CA, USA), and relative protein integrity and purity were determined by SDS/PAGE and Colloidal Blue staining (Invitrogen, Carlsbad, CA, USA). 10 μL nickel beads (with purified recombinant protein) was incubated in 1 mL of buffer for 2 h at 4 °C with *in vitro* translated proteins made using the TNT‐coupled reticulocyte *in vitro* translation system (Promega, Madison, WI, USA). Beads were washed ×10 with 1 mL of buffer. Proteins were separated by SDS/PAGE and associated proteins were detected by western blotting [48].

The NOTCH3‐ECD protein was produced using PEC.3.30, which is modified Per.E2A cells. DMEM cell supernatant containing the NOTCH3‐ECD protein was collected for purification. The supernatant was loaded onto a HisTrap high‐performance (HP) column (Cytiva, Uppsala, Sweden) and eluted with a gradient buffer that included 500 mm imidazole, 25 mm HEPES at pH 7.5 and 500 mm NaCl. The pooled fractions were concentrated using a 50 000 molecular weight cut‐off (MWCO) centrifugal filter (Millipore, Merck KGaA, Darmstadt, Germany), resulting in a buffer of 10 mm imidazole, 25 mm HEPES at pH 7.5 and 100 mm NaCl. The concentrated protein solution was subsequently loaded onto a Superdex 200 16/600 gel filtration column (Cytiva, Uppsala, Sweden), which was equilibrated with 25 mm HEPES at pH 7.5 and 100 mm NaCl. Similar yields of purified protein were obtained across all samples. The purified proteins were snap‐frozen in liquid nitrogen immediately after purification and stored at −80 °C.

### Size exclusion chromatography with multi‐angle static light scattering (SEC‐MALS)

SEC‐MALS was performed using a miniDAWN^®^ TREOS^®^ detector, DynaPro^®^ NanoStar^®^ DLS, an Optilab differential refractometer (Wyatt Technology, Santa Barbara, CA, USA), and a 1260 Infinity II multiple wavelength absorbance detector (Agilent, Thermo Fisher, Waltham, MA, USA). The temperature‐controlled autosampler was maintained at 4 °C. Separation was carried out using a Superdex 200 column (or Superdex 75 for DLL1) 10/30 GL. Data collection and analysis were conducted with the astra 8.1.1 software (Wyatt Technology). The mobile phase consisted of 38 mm NaH_2_PO_4_, 12 mm Na_2_HPO_4_, 150 mm NaCl and 200 ppm NaN_3_ at pH 7.4, prepared in HPLC‐grade water and filtered through Durapore VVPP 0.1 μm membrane filters (Millipore). Samples were centrifuged and injected in duplicates of 30 μL, with triplicate injections of BSA used for system verification during each run. Peak detection, molar mass determination and peak statistics were performed using the astra 8.1.1 software.

### Ligand/receptor binding assay

Confluent 10‐cm dishes of 293 T cells stably expressing HA epitope‐tagged NOTCH receptors were washed with ice‐cold phosphate buffered saline (PBS)/bovine serum albumen (BSA) (1 mg·mL^−1^). Cells were stimulated with 50 μL of ligand (0.5 mg·mL^−1^) in the presence of 1.5 mL of PBS/BSA (1 mg·mL^−1^) for 1 h, with gentle shaking, at 4 °C. Cells were washed once with PBS. Cells were incubated with crosslinkers in the presence of 2 mL of ice‐cold PBS for 15 min, with gentle shaking at 4 °C: 0.27 μm disuccinimidyl suberate (DSS) (Pierce, Rockford, IL, USA) and 0.07 μm bis‐sulfosuccinimidyl suberate (BS3) (Pierce). Cells were washed once with ice‐cold detachment buffer (10 mm Tris–HCl pH 7.4, 1 mm EDTA pH 7.4, 10% glycerol), scraped off the plates in 1 mL of detachment buffer and transferred to Eppendorf tubes. Cell pellets were lysed in 1 mL of solubilization buffer (125 mm NaCl, 10 mm Tris–HCl pH 7.4, 1 mm EDTA pH 7.4, 1% Triton X‐100) on ice for 30 min and centrifuged at 13000 r.p.m. for 10 min. Supernatants were incubated with HA antibody (Covance, Princeton, NJ, USA) overnight at 4 °C. 50 μL of washed (4×) protein A/G beads (50% solution) was added, and samples were incubated for 45 min at 4 °C. The beads were washed four times with solubilization buffer. 50 μL of sample buffer was added and samples were boiled for 5–10 min prior to gel electrophoresis. These experimental procedures have been described previously [49].

### NOTCH transactivation luciferase reporter assay

Reporter assays were performed as previously described [48, 49]. Briefly, stable cell lines expressing epitope‐tagged ligands and receptors were established via infection of cells with lentiviruses harbouring the appropriate ligand/receptor cDNA followed by selection with the encoded antibiotic (puromycin or neomycin). For transactivation assays, cells co‐expressing receptor and reporter were cocultured with cells stably expressing ligand alone (to enable transactivation). Comparable results were obtained for coculture ratios of 1 : 1, 1 : 2 and 1 : 4. For each experiment, cells were seeded in triplicate in 12‐well plates. The NOTCH luciferase reporter harboured 10× Recombination Signal Binding Protein for Immunoglobulin kappa J (RBPJ) consensus DNA‐binding sites, and was cotransfected with thymidine kinase promoter‐driven Renilla luciferase control plasmid. Additionally, transfection efficiencies (routinely > 90%) were determined through visualization of cotransfected plasmid encoding the Tomato fluorescence reporter. Cells were lysed 36 h postplating, and luciferase activity was measured using a luciferase assay substrate (Promega). Luciferase activity was normalized by measuring Renilla luciferase activity (Promega). Where indicated, assays were performed in the presence or absence of 10 μm DAPT (Sigma‐Aldrich, St. Louis, MO, USA). Receptor and ligand protein levels were determined by western blotting. Experiments were performed three times. The statistical hypothesis tests in this study were performed using Student's *t*‐test. We defined *P* < 0.001 as a highly significant difference (**), *P* < 0.005 as a significant difference (*), and *P* ≥ 0.005 as not significant (n.s.).

### Surface plasmon resonance (SPR)

Peptide binding to the NOTCH3‐ECD protein using SPR was performed using a Biacore T200. The NOTCH3‐ECD protein was immobilized onto a Biacore NTA Series S Sensor Chip (Cytiva), at a concentration of 0.1 μg·μL^−1^ using Nickel‐NTA capture‐coupling, resulting in approximately 3500 RU of immobilized protein. Peptides were introduced using the ‘Eject rack’ function, followed by analysis. The buffer used for both immobilization and binding consisted of 50 mm HEPES and 150 mm NaCl at 25 °C. Binding analysis was conducted at a flow rate of 30 μL·min^−1^. The entire setup was automated using biacore software, and affinity measurements were performed using the insight Software (Cytiva) steady‐state affinity model.

### T‐ALL cell viability assays

Cells were seeded, six replicates per data point, into white 96‐well plates with a clear flat bottom in 200 μL of medium at a density of 10 000 cells·mL^−1^. The medium was supplemented with the indicated concentrations of peptides/drugs (see the Results section). The number of viable cells was determined using a Cell Titer‐Blue reagent (Promega) at 1, 3, 5 and 7 days after treatment. Absorbance readings were taken at 544 nm/590 nm (RFU) using the Victor X3 multilabel plate reader (Perkin Elmer, Villebon sur Yvette, France). Experiments were performed three times. The statistical hypothesis tests in this study were performed using Student's *t*‐test. We defined *P* < 0.001 as a highly significant difference (**), *P* < 0.005 as a significant difference (*), and *P* ≥ 0.005 as not significant (n.s.). These experimental procedures have been described previously [49].

### Cell surface labelling of endogenous NOTCH1 receptors

T‐ALL cells were grown to confluence in a 10‐cm dish. Cells were washed, on ice, 3× with PBS‐CM (PBS + 0.90 mm CaCl_2_/0.33 mm MgCl_2_). Cells were incubated with crosslinkers in 2 mL of ice‐cold PBS for 15 min, with gentle shaking at 4 °C: 0.27 μm DSS (Pierce) and 0.07 μm BS3 (Pierce). Cells were washed three times with PBS‐CM, on ice. Cells were incubated with 0.5 mg·mL^−1^ sulfo‐NHS‐SS‐Biotin in PBS‐CM for 30 min on ice. Reactions were quenched by 2× wash (5 min each, on a shaker on ice) with quenching buffer (BS‐CM + 50 mm NH_4_Cl), and washed 1× with PBS‐CM. Cells were lysed in 500 μL lysis buffer (125 mm NaCl, 10 mm Tris–HCl pH 7.4, 1 mm EDTA pH 7.4, 1% Triton X‐100) for 30 min on ice. Lysates were centrifuged at 15 000 r.p.m. for 10 min at 4 °C. Supernatants were harvested, and protein concentration was measured. Supernatants were incubated with 25 μL neutravidin‐beads (Thermo Fisher) (washed 4× with 1× lysis buffer) for 30 min (rotating at 4 °C). Beads were washed 4× with lysis buffer and samples were eluted in 2× Laemmli sample buffer for western blot analysis.

### Cell cycle analysis

Cells were incubated overnight in the presence or absence of 10 μm test or control peptide. After treatment, cells were harvested, rinsed with cold PBS and fixed in 70% ice‐cold ethanol at 4 °C for 4 h. Cells were washed with ice‐cold PBS, resuspended in a staining solution containing 0.02 mg·mL^−1^ propidium iodide (PI) and 0.2 mg·mL^−1^ RNase A in PBS, and incubated in the dark at 37 °C for 20 min. The samples were analysed using flow cytometry (BD LSRFortessa™ Cytometers, Becton Dickinson, Franklin Lakes, NJ, USA).

### Proximity ligation assay (PLA)

Cells were seeded onto 12‐mm (0.17 mm thickness) coverslips (Roth, YX03.1) placed in six‐well plates. The following day, cells were incubated for 1 h at 4 °C with recombinant human DLL1 protein at a final concentration of 2 μg·mL^−1^. After treatment, cells were fixed with 4% (v/v) formaldehyde and permeabilized with 0.2% (v/v) Triton X‐100. Duolink^®^ PLA reagents (Sigma, Saint Louis, MO, USA) were used for subsequent procedures according to the manufacturer's protocol. Briefly, cells were blocked against nonspecific binding with Duolink^®^ blocking solution for 60 min at 37 °C, followed by incubation with primary antibodies (1 : 500 dilutions of anti‐HA.11 mouse monoclonal (Covance, Princeton, NJ, USA), and anti‐HA rabbit polyclonal (Abcam, Cambridge, UK)) for 2 h at 4 °C. Cells were washed in wash buffer A and incubated with PLUS and MINUS PLA probes for 1 h at 37 °C, followed by ligation with ligase for 30 min at 37 °C. Amplification was performed using polymerase for 100 min at 37 °C. Cells were washed in wash buffer B for 10 min followed by a 2 min wash in diluted buffer B (0.1%). Finally, mounting medium with DAPI (Duolink^®^) was added and cells were imaged. Fields were randomly selected using a systematic sampling method across different regions of each slide. Image processing was performed using the zen software version 3.7.

### Zebrafish xenotransplant assay

The experiments were conducted in the Leiden University zebrafish facility, a licensed establishment for the breeding and use of experimental animals and subject to internal regulations and guidelines, stating that advice is taken from the animal welfare body to minimize suffering for all experimental animals housed at the facility. The zebrafish assays described are not considered an animal experiment under the Experiments on Animals Act (Wod, effective 2014), the applicable legislation in the Netherlands in accordance with the European guidelines (EU directive no. 2010/63/EU) regarding the protection of animals used for scientific purposes, because noneating larvae were used. Therefore, a license specific for these assays on zebrafish larvae (< 6d) was not required. Zebrafish lines were originally obtained from the Zebrafish International Resource Center (University of Oregon). The *fli1a : gfp* transgenic line produces embryos in which all endothelial cells are marked by green fluorescent protein (GFP). Coupled to their optical transparency, this enables direct visualization of angiogenesis and extra‐/intravasation of labelled tumour cells [42]. Two days postfertilization (dpf), dechorionated zebrafish embryos were anesthetized with 0.003% tricaine methanesulfonate 5 min prior to injection and placed on a wet 1.5% agarose layer in a petri dish. Cultured cells (pretreated with control or test peptide) were fluorescently labelled with Vibrant DiD (#V22887; Invitrogen) as per the manufacturer's protocol. Cells were filtered using a 40‐μm cell strainer before resuspending in 1× PBS at a density of 200 cells·nL^−1^. ~5–6 μL of sample was loaded into a borosilicate glass capillary needle (Harvard Apparatus, Holliston, MA, USA) using a microloader, and the injections were performed using a Pneumatic Picopump and a manipulator (WPI, Stevenage, UK). Approximately 400 cells were injected into the pericardial space. After implantation, the embryos were collected in a petri dish containing 1× egg water and placed at 28 °C. After 2 h, the embryos were anaesthetized and injected (into the pericardial space) with either control or test peptide (10 mm stocks). Injected embryos were maintained at 33 °C. At 6 days postinjection, the embryos were anaesthetized and image acquisition and cell quantification were performed using a Leica SP5 Stimulated Emission Depletion (STED) confocal microscope. Vibrant™ DiD is a lipidic fluorescent stain that allows cell visualization at a wavelength of 647 nm. Confocal stacks were processed for maximum intensity projections with leica software. Fluorescence images from zebrafish xenografts were analysed using the quantifish software [63]. Quantification, statistical analyses and graphic representation were performed using the graphpad prism 8 software (graphpad Software). Experiments were performed three times. The statistical hypothesis tests in this study were performed using Student's *t*‐test. We defined *P* < 0.001 as a highly significant difference (**), *P* < 0.005 as a significant difference (*), and *P* ≥ 0.005 as not significant (n.s.).

### Antibodies, recombinant proteins and drugs

Antibodies were obtained from the following sources: FLAG mouse M2 monoclonal (Sigma); anti‐HA.11 mouse monoclonal (Covance); anti‐HA rabbit polyclonal (Abcam); anti‐FLAG rabbit (Sigma); anti‐γ‐tubulin (Sigma); anti‐His (Sigma); N1 rabbit monoclonal antibody (Cell Signaling Technology, Danvers, MA, USA). Recombinant human ligands were obtained from: JAG1 protein (Cat. No: JA1‐H52H9; Acrobiosystems, Newark, DE, USA); DLL4 protein (Cat. No: DL4‐H5227; Acrobiosystems); DLL4 protein (Cat. No: ab219667; AbCam).

## Conflict of interest

David Baker has filed a patent at the Dutch Patent Office: Novel NOTCH receptor inhibitors and uses thereof. All other authors declare no conflict of interest.

## Author contributions

XL performed most of the experiments assisted by HW and L‐TW. MD and XL performed receptor biotin labelling and receptor–ligand binding assays. XL and CL performed PLA experiments. GM performed the zebrafish xenotransplant studies. MAFVG, JL, XL and HW devised and performed protein purification methods. PD offered expert advice, support and supervision. DAB conceived the project and supervised the work. DAB and XL wrote the manuscript. All authors edited and approved the manuscript.

## Data Availability

Requests for further information and resources should be directed to and will be fulfilled by the lead contact, David Baker (d.baker@lumc.nl). All unique/stable reagents generated in this study are available from the lead contact with a completed materials transfer agreement. All data reported in this paper will be shared by the lead contact upon request.

## References

[1] Gazave E , Lapébie P , Richards GS , Brunet F , Ereskovsky AV , Degnan BM , Borchiellini C , Vervoort M & Renard E (2009) Origin and evolution of the notch signalling pathway: an overview from eukaryotic genomes. BMC Evol Biol 9, 1–27. 19825158 10.1186/1471-2148-9-249 PMC2770060

[2] Siebel C & Lendahl U (2017) Notch signaling in development, tissue homeostasis, and disease. Physiol Rev 97, 1235–1294. 28794168 10.1152/physrev.00005.2017

[3] Morgan TH (1917) The theory of the gene. Am Nat 51, 513–544.

[4] Guruharsha K , Kankel MW & Artavanis‐Tsakonas S (2012) The notch signalling system: recent insights into the complexity of a conserved pathway. Nat Rev Genet 13, 654–666. 22868267 10.1038/nrg3272 PMC4369923

[5] Bray SJ (2016) Notch signalling in context. Nat Rev Mol Cell Biol 17, 722–735. 27507209 10.1038/nrm.2016.94

[6] Henrique D & Schweisguth F (2019) Mechanisms of notch signaling: a simple logic deployed in time and space. Development 146, dev172148. 30709911 10.1242/dev.172148

[7] Zhou B , Lin W , Long Y , Yang Y , Zhang H , Wu K & Chu Q (2022) Notch signaling pathway: architecture, disease, and therapeutics. Signal Transduct Target Ther 7, 95. 35332121 10.1038/s41392-022-00934-y PMC8948217

[8] Meng Y , Bo Z , Feng X , Yang X & Handford PA (2024) The notch signaling pathway: mechanistic insights in health and disease. Engineering 34, 212–232.

[9] Tveriakhina L , Schuster‐Gossler K , Jarrett SM , Andrawes MB , Rohrbach M , Blacklow SC & Gossler A (2018) The ectodomains determine ligand function in vivo and selectivity of DLL1 and DLL4 toward NOTCH1 and NOTCH2 in vitro. Elife 7, e40045. 30289388 10.7554/eLife.40045 PMC6202052

[10] Luca VC , Jude KM , Pierce NW , Nachury MV , Fischer S & Garcia KC (2015) Structural basis for Notch1 engagement of Delta‐like 4. Science 347, 847–853. 25700513 10.1126/science.1261093 PMC4445638

[11] Liu L , Wada H , Matsubara N , Hozumi K & Itoh M (2017) Identification of domains for efficient notch signaling activity in immobilized notch ligand proteins. J Cell Biochem 118, 785–796. 27639253 10.1002/jcb.25744

[12] Luca VC , Kim BC , Ge C , Kakuda S , Wu D , Roein‐Peikar M , Haltiwanger RS , Zhu C , Ha T & Garcia KC (2017) Notch‐Jagged complex structure implicates a catch bond in tuning ligand sensitivity. Science 355, 1320–1324. 28254785 10.1126/science.aaf9739 PMC5459593

[13] Sprinzak D & Blacklow SC (2021) Biophysics of notch signaling. Annu Rev Biophys 50, 157–189. 33534608 10.1146/annurev-biophys-101920-082204 PMC8105286

[14] Fortini ME (2002) γ‐Secretase‐mediated proteolysis in cell‐surface‐receptor signalling. Nat Rev Mol Cell Biol 3, 673–684. 12209127 10.1038/nrm910

[15] Mumm JS , Schroeter EH , Saxena MT , Griesemer A , Tian X , Pan D , Ray WJ & Kopan R (2000) A ligand‐induced extracellular cleavage regulates γ‐secretase‐like proteolytic activation of Notch1. Mol Cell 5, 197–206. 10882062 10.1016/s1097-2765(00)80416-5

[16] Kitagawa M (2016) Notch signalling in the nucleus: roles of mastermind‐like (MAML) transcriptional coactivators. J Biochem 159, 287–294. 26711237 10.1093/jb/mvv123

[17] Kovall RA , Gebelein B , Sprinzak D & Kopan R (2017) The canonical notch signaling pathway: structural and biochemical insights into shape, sugar, and force. Dev Cell 41, 228–241. 28486129 10.1016/j.devcel.2017.04.001 PMC5492985

[18] Xu X , Choi SH , Hu T , Tiyanont K , Habets R , Groot AJ , Vooijs M , Aster JC , Chopra R & Fryer C (2015) Insights into autoregulation of Notch3 from structural and functional studies of its negative regulatory region. Structure 23, 1227–1235. 26051713 10.1016/j.str.2015.05.001 PMC4497832

[19] Gordon WR , Vardar‐Ulu D , Histen G , Sanchez‐Irizarry C , Aster JC & Blacklow SC (2007) Structural basis for autoinhibition of notch. Nat Struct Mol Biol 14, 295–300. 17401372 10.1038/nsmb1227

[20] Kovall RA & Blacklow SC (2010) Mechanistic insights into notch receptor signaling from structural and biochemical studies. Curr Top Dev Biol 92, 31–71. 20816392 10.1016/S0070-2153(10)92002-4

[21] Gordon WR , Roy M , Vardar‐Ulu D , Garfinkel M , Mansour MR , Aster JC & Blacklow SC (2009) Structure of the Notch1‐negative regulatory region: implications for normal activation and pathogenic signaling in T‐ALL. Blood 113, 4381–4390. 19075186 10.1182/blood-2008-08-174748 PMC2676092

[22] Mansour M , Linch D , Foroni L , Goldstone A & Gale R (2006) High incidence of Notch‐1 mutations in adult patients with T‐cell acute lymphoblastic leukemia. Leukemia 20, 537–539. 16424867 10.1038/sj.leu.2404101

[23] Weng AP , Ferrando AA , Lee W , Morris JP IV , Silverman LB , Sanchez‐Irizarry C , Blacklow SC , Look AT & Aster JC (2004) Activating mutations of NOTCH1 in human T cell acute lymphoblastic leukemia. Science 306, 269–271. 15472075 10.1126/science.1102160

[24] Albertí‐Servera L , Demeyer S , Govaerts I , Swings T , De Bie J , Gielen O , Brociner M , Michaux L , Maertens J & Uyttebroeck A (2021) Single‐cell DNA amplicon sequencing reveals clonal heterogeneity and evolution in T‐cell acute lymphoblastic leukemia. Blood 137, 801–811. 32812017 10.1182/blood.2020006996 PMC7885827

[25] De Bie J , Demeyer S , Alberti‐Servera L , Geerdens E , Segers H , Broux M , De Keersmaecker K , Michaux L , Vandenberghe P & Voet T (2018) Single‐cell sequencing reveals the origin and the order of mutation acquisition in T‐cell acute lymphoblastic leukemia. Leukemia 32, 1358–1369. 29740158 10.1038/s41375-018-0127-8 PMC5990522

[26] Tyagi A , Sharma AK & Damodaran C (2020) A review on notch signaling and colorectal cancer. Cells 9, 1549. 32630477 10.3390/cells9061549 PMC7349609

[27] Wang K , Zhang Q , Li D , Ching K , Zhang C , Zheng X , Ozeck M , Shi S , Li X & Wang H (2015) PEST domain mutations in notch receptors comprise an oncogenic driver segment in triple‐negative breast cancer sensitive to a γ‐secretase inhibitor. Clin Cancer Res 21, 1487–1496. 25564152 10.1158/1078-0432.CCR-14-1348

[28] Zhu H , Zhou X , Redfield S , Lewin J & Miele L (2013) Elevated Jagged‐1 and Notch‐1 expression in high grade and metastatic prostate cancers. Am J Transl Res 5, 368–378. 23634247 PMC3633979

[29] Joutel A , Corpechot C , Ducros A , Vahedi K , Chabriat H , Mouton P , Alamowitch S , Domenga V , Cécillion M & Maréchal E (1996) Notch3 mutations in CADASIL, a hereditary adult‐onset condition causing stroke and dementia. Nature 383, 707–710. 8878478 10.1038/383707a0

[30] Li X , Zhang X , Leathers R , Makino A , Huang C , Parsa P , Macias J , Yuan JX , Jamieson SW & Thistlethwaite PA (2009) Notch3 signaling promotes the development of pulmonary arterial hypertension. Nat Med 15, 1289–1297. 19855400 10.1038/nm.2021 PMC2780347

[31] Garg V , Muth AN , Ransom JF , Schluterman MK , Barnes R , King IN , Grossfeld PD & Srivastava D (2005) Mutations in NOTCH1 cause aortic valve disease. Nature 437, 270–274. 16025100 10.1038/nature03940

[32] Andersson ER & Lendahl U (2014) Therapeutic modulation of notch signalling—are we there yet? Nat Rev Drug Discov 13, 357–378. 24781550 10.1038/nrd4252

[33] Pocock R , Farah N , Richardson SE & Mansour MR (2021) Current and emerging therapeutic approaches for T‐cell acute lymphoblastic leukaemia. Br J Haematol 194, 28–43. 33942287 10.1111/bjh.17310

[34] Majumder S , Crabtree JS , Golde TE , Minter LM , Osborne BA & Miele L (2021) Targeting notch in oncology: the path forward. Nat Rev Drug Discov 20, 125–144. 33293690 10.1038/s41573-020-00091-3

[35] Cordo' V , van der Zwet JC , Canté‐Barrett K , Pieters R & Meijerink JP (2021) T‐cell acute lymphoblastic leukemia: a roadmap to targeted therapies. Blood Cancer Discov 2, 19–31. 34661151 10.1158/2643-3230.BCD-20-0093 PMC8447273

[36] Shiraz P , Jehangir W & Agrawal V (2021) T‐cell acute lymphoblastic leukemia—current concepts in molecular biology and management. Biomedicine 9, 1621. 10.3390/biomedicines9111621 PMC8615775 34829849

[37] Fattizzo B , Rosa J , Giannotta JA , Baldini L & Fracchiolla NS (2020) The physiopathology of T‐cell acute lymphoblastic leukemia: focus on molecular aspects. Front Oncol 10, 273. 32185137 10.3389/fonc.2020.00273 PMC7059203

[38] Shi Q , Xue C , Zeng Y , Yuan X , Chu Q , Jiang S , Wang J , Zhang Y , Zhu D & Li L (2024) Notch signaling pathway in cancer: from mechanistic insights to targeted therapies. Signal Transduct Target Ther 9, 128. 38797752 10.1038/s41392-024-01828-x PMC11128457

[39] Liu H , Chi AWS , Arnett KL , Chiang MY , Xu L , Shestova O , Wang H , Li YM , Bhandoola A & Aster JC (2010) Notch dimerization is required for leukemogenesis and T‐cell development. Genes Dev 24, 2395–2407. 20935071 10.1101/gad.1975210 PMC2964750

[40] Arnett KL , Hass M , McArthur DG , Ilagan MXG , Aster JC , Kopan R & Blacklow SC (2010) Structural and mechanistic insights into cooperative assembly of dimeric notch transcription complexes. Nat Struct Mol Biol 17, 1312–1317. 20972443 10.1038/nsmb.1938 PMC3024583

[41] Kobia FM , Preusse K , Dai Q , Weaver N , Hass MR , Chaturvedi P , Stein SJ , Pear WS , Yuan Z & Kovall RA (2020) Notch dimerization and gene dosage are important for normal heart development, intestinal stem cell maintenance, and splenic marginal zone B‐cell homeostasis during mite infestation. PLoS Biol 18, e3000850. 33017398 10.1371/journal.pbio.3000850 PMC7561103

[42] Somasagara RR , Huang X , Xu C , Haider J , Serody JS , Armistead PM & Leung T (2021) Targeted therapy of human leukemia xenografts in immunodeficient zebrafish. Sci Rep 11, 5715. 33707624 10.1038/s41598-021-85141-5 PMC7952715

[43] Li K , Li Y , Wu W , Gordon WR , Chang DW , Lu M , Scoggin S , Fu T , Vien L & Histen G (2008) Modulation of notch signaling by antibodies specific for the extracellular negative regulatory region of NOTCH3. J Biol Chem 283, 8046–8054. 18182388 10.1074/jbc.M800170200

[44] Tiyanont K , Wales TE , Siebel CW , Engen JR & Blacklow SC (2013) Insights into Notch3 activation and inhibition mediated by antibodies directed against its negative regulatory region. J Mol Biol 425, 3192–3204. 23747483 10.1016/j.jmb.2013.05.025 PMC3751422

[45] Ellisen LW , Bird J , West DC , Soreng AL , Reynolds TC , Smith SD & Sklar J (1991) TAN‐1, the human homolog of the drosophila notch gene, is broken by chromosomal translocations in T lymphoblastic neoplasms. Cell 66, 649–661. 1831692 10.1016/0092-8674(91)90111-b

[46] Whiteley AE , Price TT , Cantelli G & Sipkins DA (2021) Leukaemia: a model metastatic disease. Nat Rev Cancer 21, 461–475. 33953370 10.1038/s41568-021-00355-z PMC8722462

[47] Kelly DF , Lake RJ , Middelkoop TC , Fan H‐Y , Artavanis‐Tsakonas S & Walz T (2010) Molecular structure and dimeric organization of the notch extracellular domain as revealed by electron microscopy. PLoS One 5, e10532. 20479883 10.1371/journal.pone.0010532 PMC2866536

[48] Chen D , Forghany Z , Liu X , Wang H , Merks RM & Baker DA (2023) A new model of notch signalling: control of notch receptor cis‐inhibition via notch ligand dimers. PLoS Comput Biol 19, e1010169. 36668673 10.1371/journal.pcbi.1010169 PMC9891537

[49] Chen D , Liu X , Wang H , Merks RM & Baker DA (2025) A model of notch signalling control of angiogenesis: evidence of a role for notch ligand heterodimerization. PLoS Comput Biol 21, e1012825. 39932958 10.1371/journal.pcbi.1012825 PMC11841921

[50] Aster JC , Pear WS & Blacklow SC (2017) The varied roles of notch in cancer. Annual Review of Pathology: Mechanisms of Disease 12, 245–275. 10.1146/annurev-pathol-052016-100127 PMC5933931 27959635

[51] Kuhnert F , Kirshner JR & Thurston G (2011) Dll4‐notch signaling as a therapeutic target in tumor angiogenesis. Vasc Cell 3, 1–8. 21923938 10.1186/2045-824X-3-20 PMC3195111

[52] Chung AS & Ferrara N (2011) Developmental and pathological angiogenesis. Annu Rev Cell Dev Biol 27, 563–584. 21756109 10.1146/annurev-cellbio-092910-154002

[53] Carmeliet P & Jain RK (2011) Molecular mechanisms and clinical applications of angiogenesis. Nature 473, 298–307. 21593862 10.1038/nature10144 PMC4049445

[54] Herbert SP & Stainier DY (2011) Molecular control of endothelial cell behaviour during blood vessel morphogenesis. Nat Rev Mol Cell Biol 12, 551–564. 21860391 10.1038/nrm3176 PMC3319719

[55] Adams RH & Alitalo K (2007) Molecular regulation of angiogenesis and lymphangiogenesis. Nat Rev Mol Cell Biol 8, 464–478. 17522591 10.1038/nrm2183

[56] Sainson RC & Harris AL (2007) Anti‐Dll4 therapy: can we block tumour growth by increasing angiogenesis? Trends Mol Med 13, 389–395. 17822956 10.1016/j.molmed.2007.07.002

[57] Wang Z , Li Y , Kong D & H Sarkar F (2010) The role of notch signaling pathway in epithelial‐mesenchymal transition (EMT) during development and tumor aggressiveness. Curr Drug Targets 11, 745–751. 20041844 10.2174/138945010791170860 PMC3084452

[58] Kar R , Jha NK , Jha SK , Sharma A , Dholpuria S , Asthana N , Chaurasiya K , Singh VK , Burgee S & Nand P (2019) A “NOTCH” deeper into the epithelial‐to‐mesenchymal transition (EMT) program in breast cancer. Genes 10, 961. 31766724 10.3390/genes10120961 PMC6947643

[59] Meisel CT , Porcheri C & Mitsiadis TA (2020) Cancer stem cells, quo vadis? The notch signaling pathway in tumor initiation and progression. Cells 9, 1879. 32796631 10.3390/cells9081879 PMC7463613

[60] Canalis E & Zanotti S (2016) Hajdu‐Cheney syndrome, a disease associated with NOTCH2 mutations. Curr Osteoporos Rep 14, 126–131. 27241678 10.1007/s11914-016-0311-6 PMC4927394

[61] Roukens MG , Alloul‐Ramdhani M , Baan B , Kobayashi K , Peterson‐Maduro J , Van Dam H , Schulte‐Merker S & Baker DA (2010) Control of endothelial sprouting by a Tel–CtBP complex. Nat Cell Biol 12, 933–942. 20835243 10.1038/ncb2096

[62] Forghany Z , Robertson F , Lundby A , Olsen JV & Baker DA (2018) Control of endothelial cell tube formation by notch ligand intracellular domain interactions with activator protein 1 (AP‐1). J Biol Chem 293, 1229–1242. 29196606 10.1074/jbc.M117.819045 PMC5787801

[63] Stirling DR , Suleyman O , Gil E , Elks PM , Torraca V , Noursadeghi M & Tomlinson GS (2020) Analysis tools to quantify dissemination of pathology in zebrafish larvae. Sci Rep 10. 10.1038/s41598-020-59932-1 PMC7035342 32081863

